# Running, walking, and cross-country skiing: how to shape adolescents’ personalities through physical activity?

**DOI:** 10.3389/fpsyg.2024.1489131

**Published:** 2024-11-13

**Authors:** Yucheng Gao, Li Che, Xiaotian Li

**Affiliations:** ^1^Institute of Physical Education and Training, Capital University of Physical Education and Sports, Beijing, China; ^2^School of Dance and Martial Arts, Capital University of Physical Education and Sports, Beijing, China; ^3^School of Recreation and Community Sport, Capital University of Physical Education and Sports, Beijing, China

**Keywords:** adolescent personality, sports participation, Big Five Personality Traits, gender differences, physical activity frequency, Seemingly Unrelated Regression Models

## Abstract

Adolescence is crucial for personality development, and sports play a significant role. This study investigates the impact of various sports on the personality traits of junior high and high school students in Shandong Province, focusing on neuroticism, extraversion, openness, agreeableness, and conscientiousness. Utilizing data from the “Database of Youth Health,” we employed Seemingly Unrelated Regression (SUR) and Generalized Structural Equation Modeling (GSEM) to analyze the effects of physical activity on personality development. Findings reveal that walking significantly enhances openness and decreased neuroticism, while jogging/running substantially improves extraversion, agreeableness, and conscientiousness. Cross-country skiing, however, negatively impacts all assessed personality traits. In addition, the importance of gender differences in the relationship between physical activity and personality development was revealed. The results offer insights for promoting adolescent personality development through targeted sports activities.

## 1 Introduction

Adolescence is a critical phase of rapid physical and mental development, as well as a crucial period for shaping and developing personality traits (Shiner et al., 2023; Logan and Ward-Ritacco, 2022). During this stage, an individual’s personality traits not only have a profound impact on their current psychological health (Donker et al., 2024; Kumar and Vijayakumar, n.d.) but also affect their future career choices (Arslan et al., 2023), interpersonal relationships (Shiner et al., 2023), and life satisfaction (Kang and Malvaso, 2023). Therefore, exploring factors that influence the development of adolescent personality traits holds significant theoretical and practical value, especially those factors that can be altered through personal or societal intervention. Sports activities, as a common recreational activity, are widely believed to have a positive impact on psychological health and personality development (Tahira, 2022; Eather et al., 2023; Zhou et al., 2022; Kumar et al., 2023), hence the role of sports in promoting the positive development of personality traits has received extensive attention.

Existing research on the relationship between sports and personality traits is abundant. Most scholars have found that extraversion, openness, agreeableness, and conscientiousness are positively correlated with physical activity, while neuroticism is negatively correlated with physical activity (Rhodes and Smith, 2006; Jarvis, 2006; Elman and Mckelvie, 2003; Sevcikova et al., 2000; Ledwidge, 1980; Jasnoski et al., 1988; Liao et al., 2022; Allen et al., 2021; Rhodes and Wilson, 2020; Hakulinen and Jokela, 2018). However, some scholars believe that there is no correlation between physical activity and neuroticism (Stephan et al., 2018; Allen et al., 2015; Stephan et al., 2014). Further studies have delved deeper into the relationship between sports and personality traits, exploring the impact of participating in different types of sports on personality traits. For example, Schurr et al. (1977) found that team athletes were more anxious and extroverted than individual athletes; Francis et al. (1998) found that female hockey players, compared to those not formally engaged in sports, had higher levels of extraversion and neuroticism. Breivik (1996) conducted a 16 PF test on 38 elite Norwegian climbers, finding them to have very low neuroticism and higher extraversion and adventurousness; Freixanet (1991) conducted an EPQ test on a group of high-risk sports participants (including 72 mountaineers) and a group of low-risk athletes, finding that mountaineers and other high-risk sports participants had higher extraversion and lower neuroticism levels; Diehm and Armatas (2004) used NEO-PI to compare the personality traits of 44 golfers (low-risk) and 41 surfers (high-risk), finding that surfers had higher openness. Additionally, scholars have also focused on the sports level of participants. Egloff and others, and Williams and others, found that elite athletes were more extroverted and less neurotic than recreational athletes (Egloff and Gruhn, 1996; Williams and Parkin, 1980). Although existing research on personality traits and sports is relatively abundant, there are still three shortcomings that need to be addressed. First, many studies have used older personality scales, such as Cattell’s 16PF Personality Scale and the Eysenck Personality Questionnaire, which reduces the applicability of these studies’ conclusions in modern sports psychology. Second, few studies have classified sports by discipline and conducted horizontal comparisons between different sports, investigating the impact of participation in various sports on personality traits. In practice, understanding the differences between different sports can help coaches and educators develop more targeted physical activities based on individuals’ specific needs and personality development goals. Third, the sample sizes in previous studies have been relatively small (most involving around 100 participants, while this study includes 21,521), which limits the generalizability of their conclusions. In this paper, we conducted an in-depth study on the relationship between physical activity and personality traits, addressing these three shortcomings.

In this research, data derived from the “The Database of Youth Health” were utilized to focus on junior high and high school students across 17 cities in Shandong Province. By analyzing their sports behaviors and personality traits, the aim is to delve deeper into the impact of different sports on the development of adolescents’ personality traits and to examine whether gender plays a moderating role in this relationship. The main variables of interest in this study are the five personality traits from the Big Five personality model—neuroticism, extraversion, openness, agreeableness, and conscientiousness—as well as various sports activities participated in by the adolescents, such as jumping rope, roller skating, tag games, and walking exercises. Through the analysis of Seemingly Unrelated Regression Models, this study not only explores the impact of participating in specific sports on personality traits but also assesses the moderating effect of the frequency of sports activities on this relationship. Further, given the significant plasticity of personality traits during adolescence (Belsky, 2013; Schriber and Guyer, 2016)—a critical period for psychosocial development—focusing on this group adds substantial real-world relevance to the study.

Through a systematic analysis of the relationship between adolescent sports behavior and personality traits, this study aims to provide a scientific basis for promoting adolescent personality development through sports. It also aims to offer references for parents, schools, and policymakers in the arrangement of adolescent education and sports activities. Additionally, the results of the study will enrich the theoretical knowledge in the fields of sports psychology and developmental psychology, especially in understanding the mechanisms of how sports influence the development of adolescent personality traits and the aspects of gender differences.

## 2 Data, variables, and analysis strategy

### 2.1 Data

This paper utilizes the “The Database of Youth Health” (a cross-sectional dataset) for related empirical research (Zhang et al., 2022). This project conducted multiple rounds of surveys in 2015, 2016, 2017, and 2020 among junior high and high school students in 17 cities of Shandong Province. The survey covered 11 aspects, including personal information, family background, school adaptation, sports behavior, among others (not all 11 aspects were included in every round of the survey). We used Stata software to filter the dataset, retaining only the samples that contained all four aspects (including the variables relevant to our research)—personal information, family background, school adaptation, and sports behavior. This resulted in 23,314 samples. After excluding 1,793 samples that contained missing or anomalous values (those outside the specified range), 21,521 samples were ultimately included in the empirical analysis model. Descriptive statistics of the data and variables are shown in Table 1.

**Table 1:** Descriptive statistics for key variables.

Variable	Mean	Standard deviation	Minimum	Maximum
Neuroticism	4.302	2.082	3	15
Extraversion	10.214	2.539	3	14
Openness (negative scoring)	3.341	1.367	2	9
Agreeableness	24.360	4.876	7	32
Conscientiousness	21.608	4.533	6	29
Jump rope	2.330	1.348	1	5
Roller skating	1.672	1.158	1	5
Chase and capture game	2.022	1.223	1	5
Walking exercise	3.189	1.399	1	5
Cycling	2.358	1.439	1	5
Jogging or running	2.950	1.233	1	5
Swimming	1.548	1.045	1	5
Baseball, softball	1.437	0.964	1	5
Dance-based movement	1.580	1.072	1	5
Badminton	1.998	1.241	1	5
Skateboarding	1.597	1.089	1	5
Soccer	1.785	1.165	1	5
Volleyball	1.637	1.099	1	5
Basketball	2.014	1.290	1	5
Ice skating	1.463	0.999	1	5
Cross-country skiing	1.373	0.913	1	5
Other	1.891	1.201	1	5

### 2.2 Variables

#### 2.2.1 Dependent variables

The dependent variables in this paper are the five personality traits from the Big Five personality model: neuroticism, extraversion, openness, agreeableness, and conscientiousness. Based on the definitions and descriptions of these dimensions by multiple scholars (Costa and Mccrae, 1992; John, 1990; John and Srivastava, 1999; Soto and John, n.d.; Saucier and Ostendorf, 1999), the author selected corresponding questions from the “The Database of Youth Health” to measure the development levels of these personality traits among middle school students. Table 2 displays the test items for each personality trait dimension. To further assess the internal consistency of the Big Five Personality Traits Scale, this study conducted a Cronbach’s Alpha reliability test on 21 items. The results showed that the Cronbach’s alpha coefficient of the scale was 0.907, indicating that the scale has a very high internal consistency. This result suggests that the 21 items selected were able to reliably and consistently measure the Big Five Personality Traits of the subjects. In this study, the test items for openness are scored negatively, meaning that lower scores on openness indicate higher developmental levels of this personality trait.

**Table 2:** Big Five personality dimensions test items.

Personality traits	Description and definition	Measurement item
Openness	Ideas; Actions; Feelings; Values (Costa and Mccrae, 1992); Narrow interests; Simple; Shallow (John, 1990); Conservative attitudes (John and Srivastava, 1999)	1. Do you often find life uninteresting? 2. Unwilling to share with other students
Conscientiousness	Order; Dutifulness; Self~Discipline; Competence; Deliberation (Costa and Mccrae, 1992); Organized; Reliable; Painstaking (John, 1990); following norms and rules (John and Srivastava, 1999)	1. Have the ability to be admired by classmates 2. Complete assigned homework or tasks on time 3. Follows classroom discipline 4. The ability to adapt to the demands and expectations of school 5. Have the ability to exercise self-control and self-discipline 6. When faced with a difficult task, can you still keep doing it?
Extraversion	Talkative; Assertive; Outspoken; Dominant; Sociable (John, 1990); number of friends and sex partners (John and Srivastava, 1999); number of friends (John and Srivastava, 1999)	1. Show decisiveness when needed 2. Do you have many good friends? 3. Skillfully interacts with or joins in with classmates 4. Socialize with many peers
Agreeableness	Sympathetic; Kind; Affectionate; Soft-hearted; Generous; Trusting; Helpful (John, 1990); Trust; Altruism; Empathy (Soto and John, n.d.); Emphasize the good qualities of other people when I talk about them (John and Srivastava, 1999)	1. Be able to take the initiative to help classmates 2. Understand the problems and needs of classmates 3. Be able to recognize the psychological changes of other students. 4. Be able to avoid losing my temper when I am angry or furious. 5. Do you think your classmates are friendly to you? 6. Do your friends care about you? 7. Do you think your teachers are friendly to you?
Neuroticism	Moody; Temperamental; Unstable; Emotional (John, 1990); Emotionality; Irritability (Soto and John, n.d.; Saucier and Ostendorf, 1999); Impulsiveness (Costa and Mccrae, 1992)	1. Easily provoked and easily offended 2. Behavior is difficult to control 3. Behaves impulsively without thinking

#### 2.2.2 Independent variables

The main independent variables in this paper are the frequencies of participation in different types of sports. The corresponding question in the “Adolescent Health Themes Database” survey is: “How often did you participate in the following sports during leisure time in the past 7 days?” There are 18 categories of sports listed under this question. However, since “aerobic exercises” include activities like cycling and jogging, which could cause multicollinearity issues, aerobic exercises are not included in this study. As shown in Table 1, there are 17 main independent variables in this study, corresponding to the participation frequencies in 17 different types of sports, with values ranging from 1 to 5, where “1” represents none, never done; “2” represents 1–2 times; “3” represents 3–4 times; “4” represents 5–6 times; “5” represents 7 times or more.

#### 2.2.3 Control variables

After reviewing a substantial amount of literature (Sherafati et al., 2020; Imam et al., 2021; Kekäläinen et al., 2020; Markowska et al., 2017; Modestin, 2006; Nakao et al., 2000; Anderson et al., 2024; Milenkova and Nakova, 2023), we selected a number of factors from the database that may influence sports participation (independent variable) and personality development (dependent variable) as control variables. These include gender, place of residence, mother’s education level, father’s education level, family economic condition, father’s occupation, mother’s occupation, the frequency of vigorous physical activity during physical education classes in the past 7 days, and family relationships.

### 2.3 Analysis strategy

The dependent variables in this paper are five in number, and the independent variables used to estimate these five personality traits are identical, which means that any omitted variables not included in the regression will affect all five dependent variables simultaneously. Additionally, there is likely to be a correlation among the five personality traits, and using five separate OLS models for regression would result in inaccurate estimates of the coefficients of the independent variables. Therefore, we choose to use the Seemingly Unrelated Regression (SUR) model for estimation. The SUR model takes into account the correlation among the dependent variables of the five independent models, allowing for joint estimation, which can also improve the estimation bias caused by omitted variables, thereby enhancing the estimation efficiency of the model.

## 3 Empirical analysis of the impact of participating in different sports on personality traits

### 3.1 Which sports most significantly enhance adolescent personality development?

**Figure 1:**
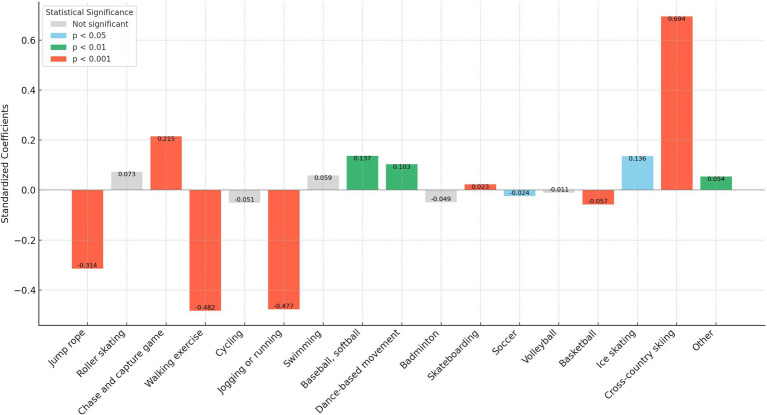
Impact of different sports on the neuroticism trait.

**Figure 2:**
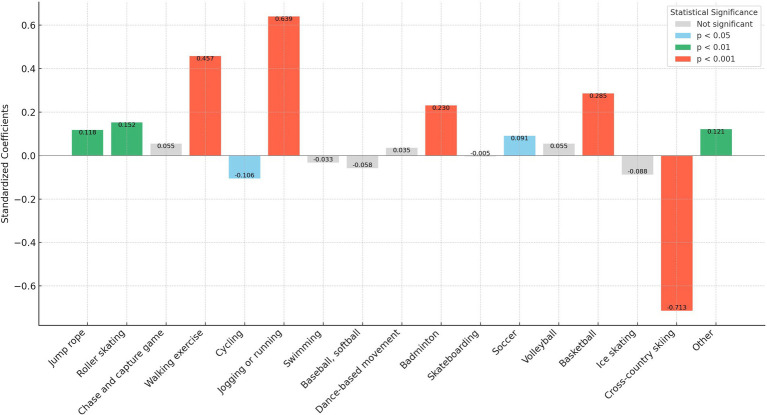
Impact of different sports on the extraversion trait.

**Figure 3:**
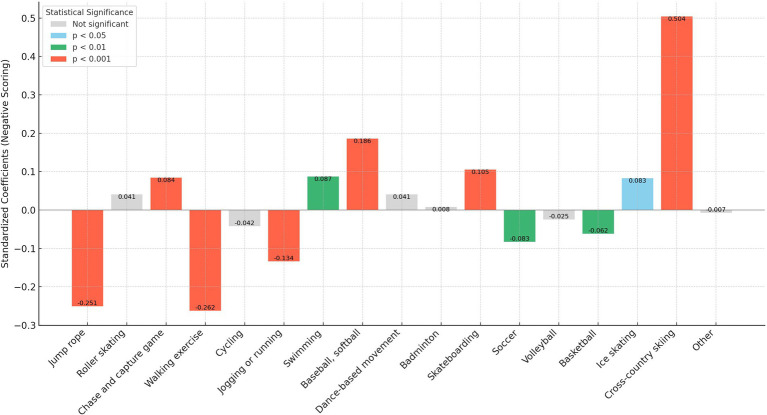
Impact of different sports on the openness trait.

**Figure 4:**
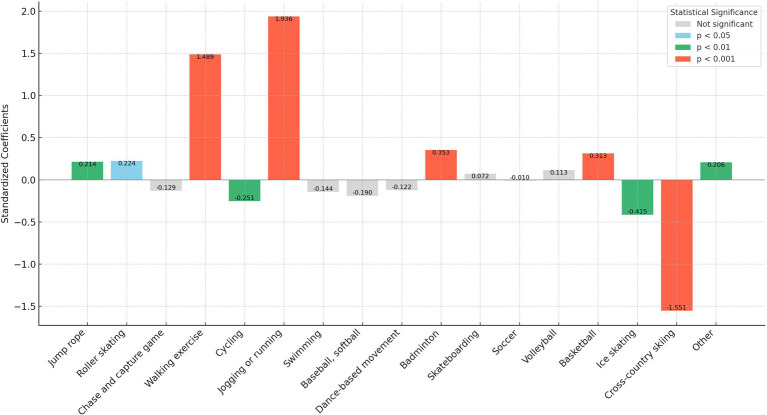
Impact of different sports on the agreeableness trait.

**Figure 5:**
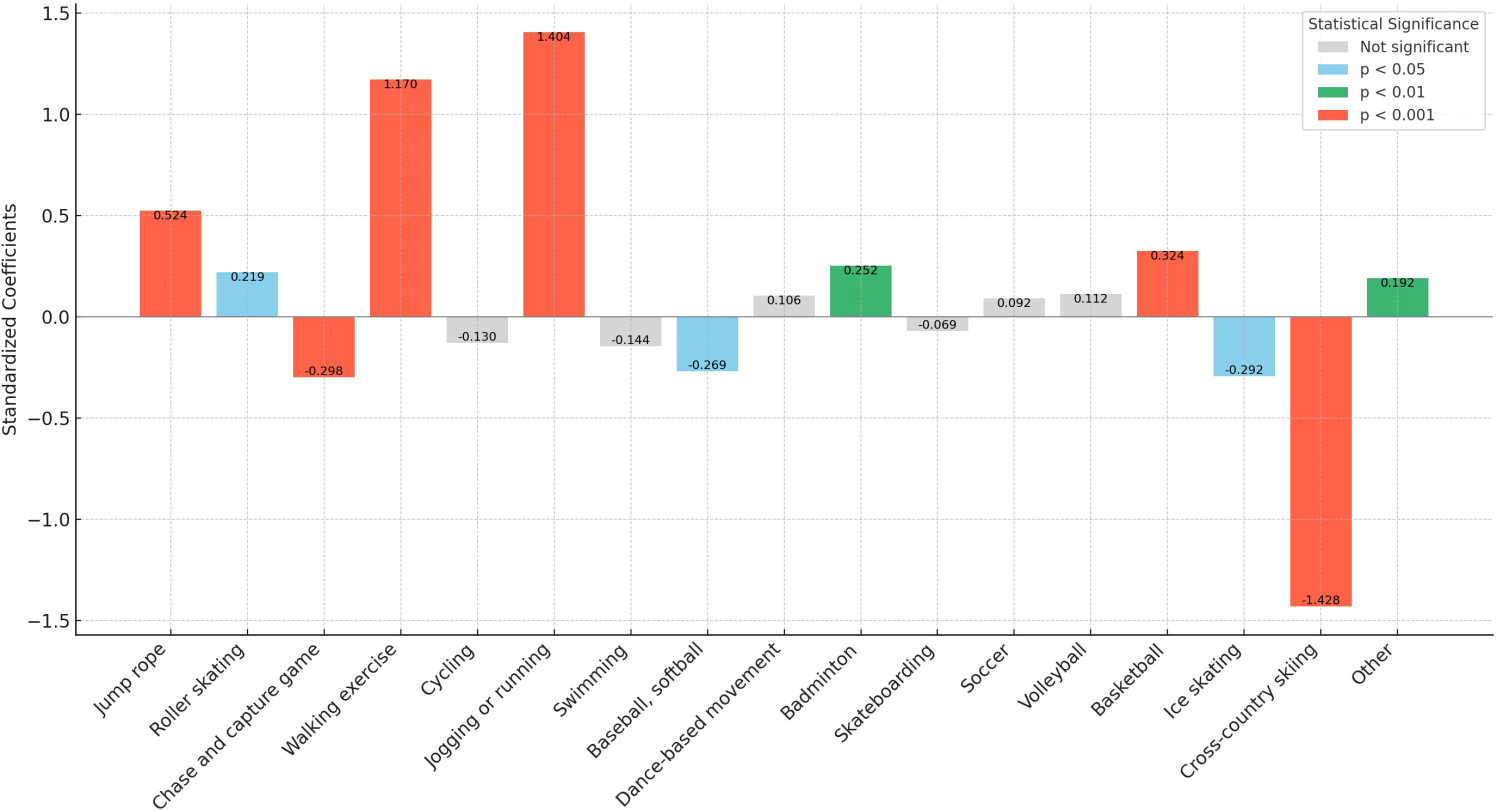
Impact of different sports on the conscientiousness trait.

In our analysis, we transformed the independent variables representing sports participation frequency into binary variables. Here, a frequency of zero times per week was encoded as “0,” and any frequency above zero was encoded as “1.” This binary recoding was implemented to more straightforwardly assess and compare the marginal effects of different sports on personality traits. This approach simplifies the statistical analysis, allowing clearer interpretations of how even minimal participation in various sports influences personality traits compared to non-participation. In addition, as shown in Figures 1–5, we visualized the results of the data analysis (here the results of the data analysis controlled for control variables) in order to present the results of the study in a more intuitive way.

The analysis conducted using Seemingly Unrelated Regression Models, coupled with the specific criteria of the test items for each personality dimension, identifies optimal sports for personality development. Walking exercise is most effective in reducing neuroticism (*B* = −0.482, *p* < 0.001), while jogging or running excels in fostering extraversion (*B* = 0.639, *p* < 0.001), agreeableness (*B* = 1.963, *p* < 0.001), and conscientiousness (*B* = 1.404, *p* < 0.001). Walking exercise also promotes openness (*B* = −0.262, *p* < 0.001). Figures 1–5 further illustrate that both walking and jogging or running significantly enhance all five personality traits.

Furthermore, as indicated in Table 3, the ranking of the best sports for impacting various personality traits remains consistent, regardless of the inclusion of control variables in the regression analyses. This consistency underscores the robustness of the findings, suggesting that the identified relationships are stable across different model specifications.

**Table 3:** Effects of participation in different sports on different personality traits.

	Model 1					Model 2				
	Neuroticism	Extraversion	Openness (negative scoring)	Agreeableness	Conscientiousness	Neuroticism	Extraversion	Openness (negative scoring)	Agreeableness	Conscientiousness
Jump rope	−0.314^***^ (0.034)	0.118^**^ (0.042)	−0.251^***^ (0.023)	0.214^**^ (0.078)	0.524^***^ (0.073)	−0.477^***^ (0.035)	0.301^**^ (0.043)	−0.344^***^ (0.023)	0.681^***^ (0.081)	0.919^***^ (0.076)
Roller skating	0.073 (0.039)	0.152^**^ (0.049)	0.041 (0.026)	0.224^*^ (0.090)	0.219^*^ (0.085)	0.128^**^ (0.041)	0.095 (0.051)	0.075^**^ (0.027)	0.097 (0.095)	0.106 (0.090)
Chase and capture game	0.215^***^ (0.030)	0.055 (0.038)	0.084^***^ (0.020)	−0.129 (0.069)	−0.298^***^ (0.066)	0.240^***^ (0.032)	0.035 (0.039)	0.096^***^ (0.021)	−0.182^*^ (0.073)	−0.345^***^ (0.069)
Walking exercise	−0.482^***^ (0.049)	0.457^***^ (0.060)	−0.262^***^ (0.032)	1.489^***^ (0.111)	1.170^***^ (0.105)	−0.667^***^ (0.050)	0.683^***^ (0.062)	−0.367^***^ (0.033)	2.049^***^ (0.117)	1.650^***^ (0.110)
Cycling	−0.051 (0.034)	−0.106^*^ (0.041)	−0.042 (0.022)	−0.251^**^ (0.077)	−0.130 (0.072)	−0.074^*^ (0.035)	−0.064 (0.043)	−0.058^*^ (0.023)	−0.210^**^ (0.081)	−0.074 (0.076)
Jogging or running	−0.477^***^ (0.046)	0.639^***^ (0.057)	−0.134^***^ (0.031)	1.936^***^ (0.105)	1.404^***^ (0.010)	0.662^***^ (0.048)	0.867^***^ (0.059)	−0.224^***^ (0.031)	2.454^***^ (0.111)	1.834^***^ (0.104)
Swimming	0.059 (0.044)	−0.033 (0.054)	0.087^**^ (0.029)	−0.144 (0.100)	−0.144 (0.095)	0.075 (0.045)	0.015 (0.056)	0.080^**^ (0.030)	−0.077 (0.106)	−0.047 (0.010)
Baseball, softball	0.137^**^ (0.053)	−0.058 (0.065)	0.186^***^ (0.035)	−0.190 (0.120)	−0.269^*^ (0.113)	0.207^***^ (0.054)	−0.144^*^ (0.067)	0.236^***^ (0.036)	−0.443^***^ (0.127)	−0.483^***^ (0.119)
Dance-based movement	0.103^**^ (0.039)	0.035 (0.048)	0.041 (0.026)	−0.122 (0.089)	0.106 (0.084)	0.056 (0.039)	0.021 (0.048)	0.004 (0.026)	0.102 (0.091)	0.231^**^ (0.085)
Badminton	−0.049 (0.034)	0.230^***^ (0.042)	0.008 (0.022)	0.353^***^ (0.077)	0.252^**^ (0.073)	−0.045 (0.035)	0.211^***^ (0.043)	0.024 (0.023)	0.279^**^ (0.081)	0.172^*^ (0.076)
Skateboarding	0.023 (0.043)	−0.005 (0.053)	0.105^***^ (0.029)	0.072 (0.099)	−0.069 (0.093)	0.019 (0.045)	−0.019 (0.056)	0.104^***^ (0.030)	0.065 (0.105)	−0.88 (0.098)
Soccer	−0.024 (0.037)	0.091^*^ (0.046)	−0.083^**^ (0.025)	−0.0104 (0.085)	0.092 (0.080)	0.019 (0.038)	0.101^*^ (0.047)	−0.061^*^ (0.025)	−0.234^**^ (0.089)	0.027 (0.084)
Volleyball	−0.011 (0.039)	0.055 (0.048)	−0.025 (0.026)	0.113 (0.089)	0.112 (0.084)	−0.022 (0.040)	0.074 (0.050)	−0.036 (0.026)	0.210^*^ (0.094)	0.191^*^ (0.088)
Basketball	−0.057 (0.035)	0.285^***^ (0.043)	−0.062^**^ (0.023)	0.313^***^ (0.080)	0.324^***^ (0.075)	−0.033 (0.035)	0.363^***^ (0.044)	−0.049^*^ (0.023)	0.257^**^ (0.082)	0.357^***^ (0.077)
Ice skating	0.136^*^ (0.053)	−0.088 (0.066)	0.083^*^ (0.025)	−0.415^**^ (0.122)	−0.292^*^ (0.115)	0.167^**^ (0.055)	−0.139^*^ (0.068)	0.106^**^ (0.036)	−0.512^***^ (0.129)	−0.393^**^ (0.121)
Cross-country skiing	0.694^***^ (0.056)	−0.713^***^ (0.069)	0.504^***^ (0.037)	−1.551^***^ (0.128)	−1.428^***^ (0.121)	0.838^***^ (0.058)	−0.853^***^ (0.072)	0.567^***^ (0.038)	−1.870^***^ (0.135)	−1.685^***^ (0.127)
Cross-country skiing	0.054 (0.030)	0.121^**^ (0.037)	−0.007 (0.020)	0.206^**^ (0.068)	0.192^**^ (0.064)	0.018 (0.031)	0.176^***^ (0.038)	−0.029 (0.020)	0.323^***^ (0.071)	0.295^**^ (0.067)
Feature control	Yes	Yes	Yes	Yes	Yes	No	No	No	No	No

The control variables include gender, place of residence, parents’ education levels, parents’ occupations, family economic conditions, the frequency of vigorous exercise during physical education classes in the past 7 days, and family economic relationships. “*” indicates *p* < 0.05, “**” indicates *p* < 0.01, “***” indicates *p* < 0.001. In the personality traits, openness is scored negatively, meaning that a lower openness score indicates better development of openness traits. In each column, the coefficient with the largest absolute value that is significant is marked with “underline” (only considering sports activities that contribute to positive personality development). The coefficients shown in parentheses are standard errors.

### 3.2 Robustness tests

#### 3.2.1 Addressing changes in model error structure

**Figure 6:**
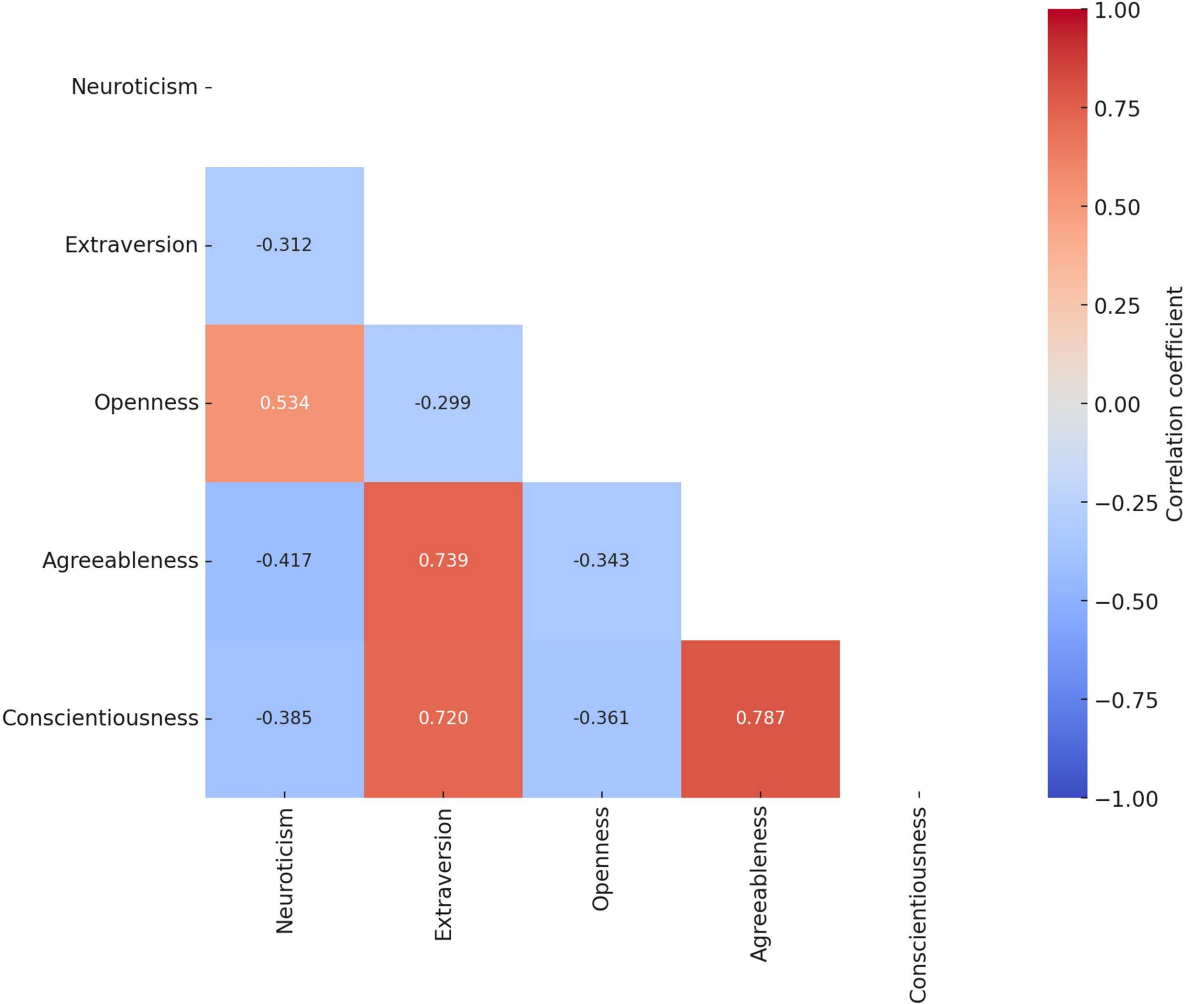
Pearson correlation coefficients between personality traits.

We used Pearson correlation analysis to test the correlation between the dependent variables in this paper, as shown in Figure 6, and found that there is a high correlation between all five dependent variables studied in this paper, indicating that there is a need to use seemingly unrelated regression (SUR) for joint estimation to improve the efficiency of model estimation.

#### 3.2.2 Modifying the estimation approach

To ensure that the findings of this study are not disproportionately influenced by a specific estimation technique, thereby potentially compromising robustness, the author employed Generalized Structural Equation Modeling (GSEM). This method is adept at elucidating complex interactions between observed and latent variables. Additionally, robust standard errors were computed to refine the accuracy of the parameter estimates. According to the results displayed in Table 4, the outcomes using GSEM align perfectly with those from the Seemingly Unrelated Regression model, with no change in the sports identified as most beneficial for each personality trait. This consistency across different estimation methods reaffirms the robustness of the study’s results, demonstrating that the findings are not uniquely tied to any single regression technique.

**Table 4:** Effects of participation in different sports on different personality traits.

	Neuroticism	Extraversion	Openness (negative scoring)	Agreeableness	Conscientiousness
Jump rope	−0.314^***^ (0.032)	0.118^**^ (0.041)	−0.251^***^ (0.022)	0.214^**^ (0.075)	0.524^***^ (0.070)
Roller skating	0.073^*^ (0.037)	0.152^**^ (0.048)	0.041 (0.025)	0.224^*^ (0.089)	0.219^**^ (0.083)
Chase and capture game	0.215^***^ (0.026)	0.055 (0.036)	0.084^***^ (0.019)	−0.129^*^ (0.066)	−0.298^***^ (0.061)
Walking exercise	−0.482^***^ (0.055)	0.457^***^ (0.063)	−0.262^***^ (0.035)	1.489^***^ (0.119)	1.170^***^ (0.112)
Cycling	−0.051 (0.030)	−0.106^**^ (0.041)	−0.042^*^ (0.021)	−0.251^**^ (0.072)	−0.130 (0.067)
Jogging or running	−0.477^***^ (0.051)	0.639^***^ (0.059)	−0.134^***^ (0.032)	1.936^***^ (0.112)	1.404^***^ (0.104)
Swimming	0.059 (0.042)	−0.033 (0.053)	0.087^**^ (0.029)	−0.144 (0.099)	−0.144 (0.092)
Baseball, softball	0.137^**^ (0.053)	−0.058 (0.064)	0.186^***^ (0.036)	−0.190 (0.121)	−0.269^*^ (0.114)
Dance-based movement	0.103^**^ (0.037)	0.035 (0.047)	0.041 (0.025)	−0.122 (0.087)	0.106 (0.081)
Badminton	−0.049 (0.030)	0.230^***^ (0.040)	0.008 (0.021)	0.353^***^ (0.072)	0.252^***^ (0.068)
Skateboarding	0.023 (0.040)	−0.005 (0.053)	0.105^***^ (0.028)	0.072 (0.096)	−0.069 (0.090)
Soccer	−0.024 (0.034)	0.091^*^ (0.044)	−0.083^**^ (0.023)	−0.104 (0.082)	0.092 (0.076)
Volleyball	−0.011 (0.035)	0.055 (0.046)	−0.025 (0.024)	0.113 (0.084)	0.112 (0.078)
Basketball	−0.057 (0.032)	0.285^***^ (0.042)	−0.062^**^ (0.022)	0.313^***^ (0.077)	0.324^***^ (0.072)
Ice skating	0.136^*^ (0.055)	−0.088 (0.064)	0.083^*^ (0.038)	−0.415^**^ (0.125)	−0.292^*^ (0.116)
Cross-country skiing	0.694^***^ (0.062)	−0.713^***^ (0.071)	0.504^***^ (0.042)	−1.551^***^ (0.137)	−1.428^***^ (0.130)
Other	0.054^*^ (0.026)	0.121^**^ (0.036)	−0.007 (0.018)	0.206^**^ (0.064)	0.192^**^ (0.060)
Feature control	Yes	Yes	Yes	Yes	Yes

Statistical significance is denoted as follows: “*” indicates *p* < 0.05, “**” indicates *p* < 0.01, and “***” indicates *p* < 0.001. The analysis controls for several variables: gender, residential location, levels of parental education, parental employment status, family economic conditions, the frequency of intense physical activity in physical education classes over the previous week, and the dynamics of family economics. Within each column of the results, the most significant coefficients—those with the largest absolute values—are highlighted with “underline,” focusing only on sports activities that foster beneficial development in personality traits.

#### 3.2.3 Modifying data sample segmentation

Given the documented differences in physiology (Burke et al., 2019), societal expectations (Çerimli, 2022; Christen, 2017), and body perception between genders (Picone et al., 2022; Breda-Vicentini et al., 2020), it is anticipated that the relationship between sports participation and personality traits might vary by gender. Consequently, it is essential to stratify the study’s sample into male and female groups to conduct distinct analyses. This approach will enable a more detailed examination of how participation in various sports affects personality traits differently across genders.

**Figure 7:**
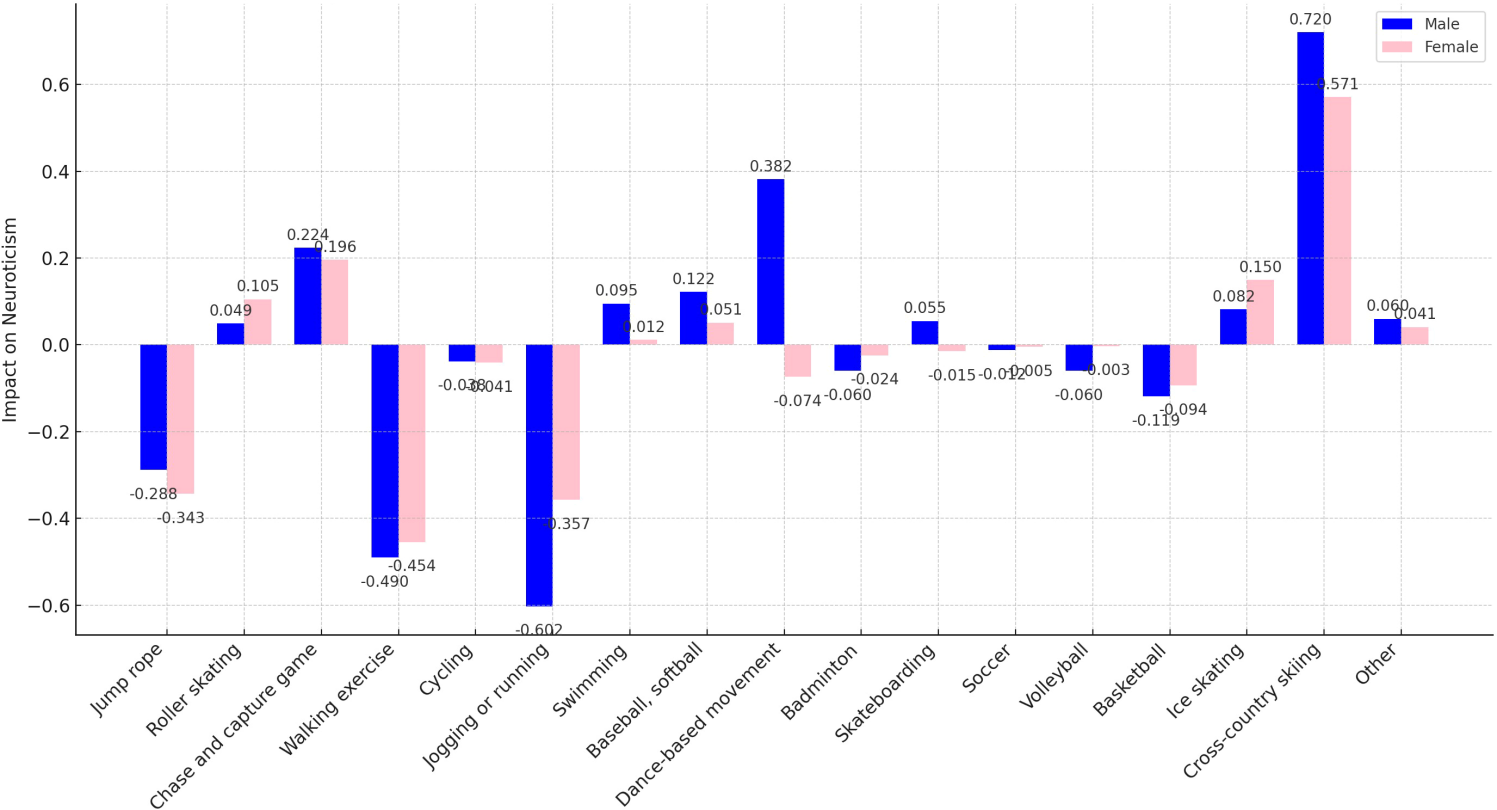
Impact of participation in different sports on neuroticism by gender.

As delineated in Figures 7, 9, the sample was categorized by gender for targeted regression analyses. The findings indicated no gender disparity in the optimal sports for enhancing extraversion and openness, with jogging or running and walking exercise proving most beneficial, respectively.

**Figure 8:**
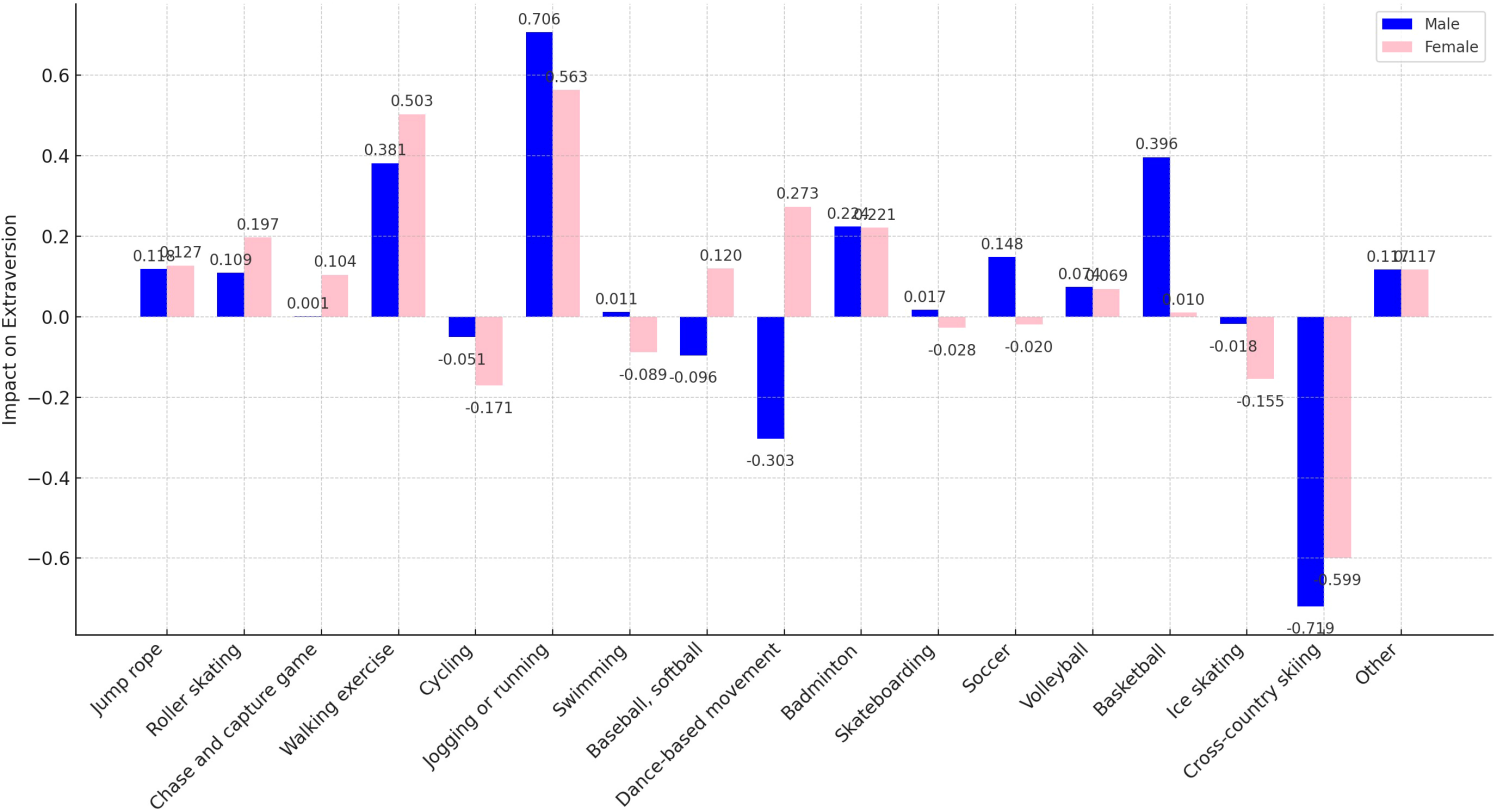
Impact of participation in different sports on openness by gender.

However, significant gender-specific differences were observed in the optimal sports for neuroticism, agreeableness, and conscientiousness, as detailed in Figures 8, 10, 11. For males, jogging or running is most effective in reducing neuroticism (*B* = −0.602, *p* < 0.001) and enhancing agreeableness (*B* = 2.196, *p* < 0.001) and conscientiousness (*B* = 1.665, *p* < 0.001). For females, walking exercise yields the best results in lowering neuroticism (*B* = −0.454, *p* < 0.001), improving agreeableness (*B* = 1.724, *p* < 0.001), and boosting conscientiousness (*B* = 1.258, *p* < 0.001). These outcomes underscore the importance of considering gender when recommending sports activities for personality development.

**Figure 9:**
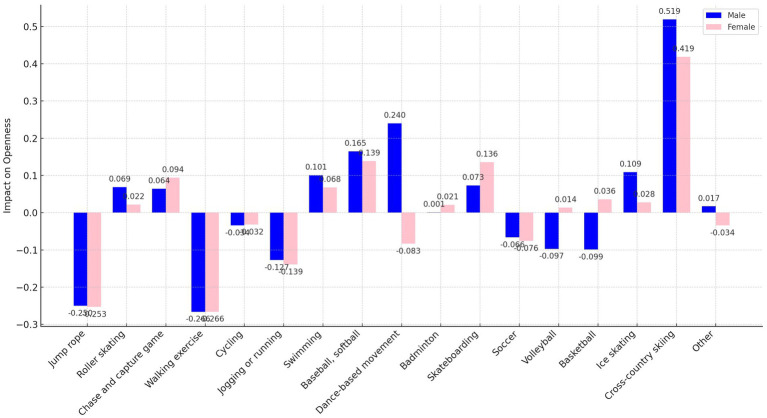
Impact of participation in different sports on extraversion by gender.

**Figure 10:**
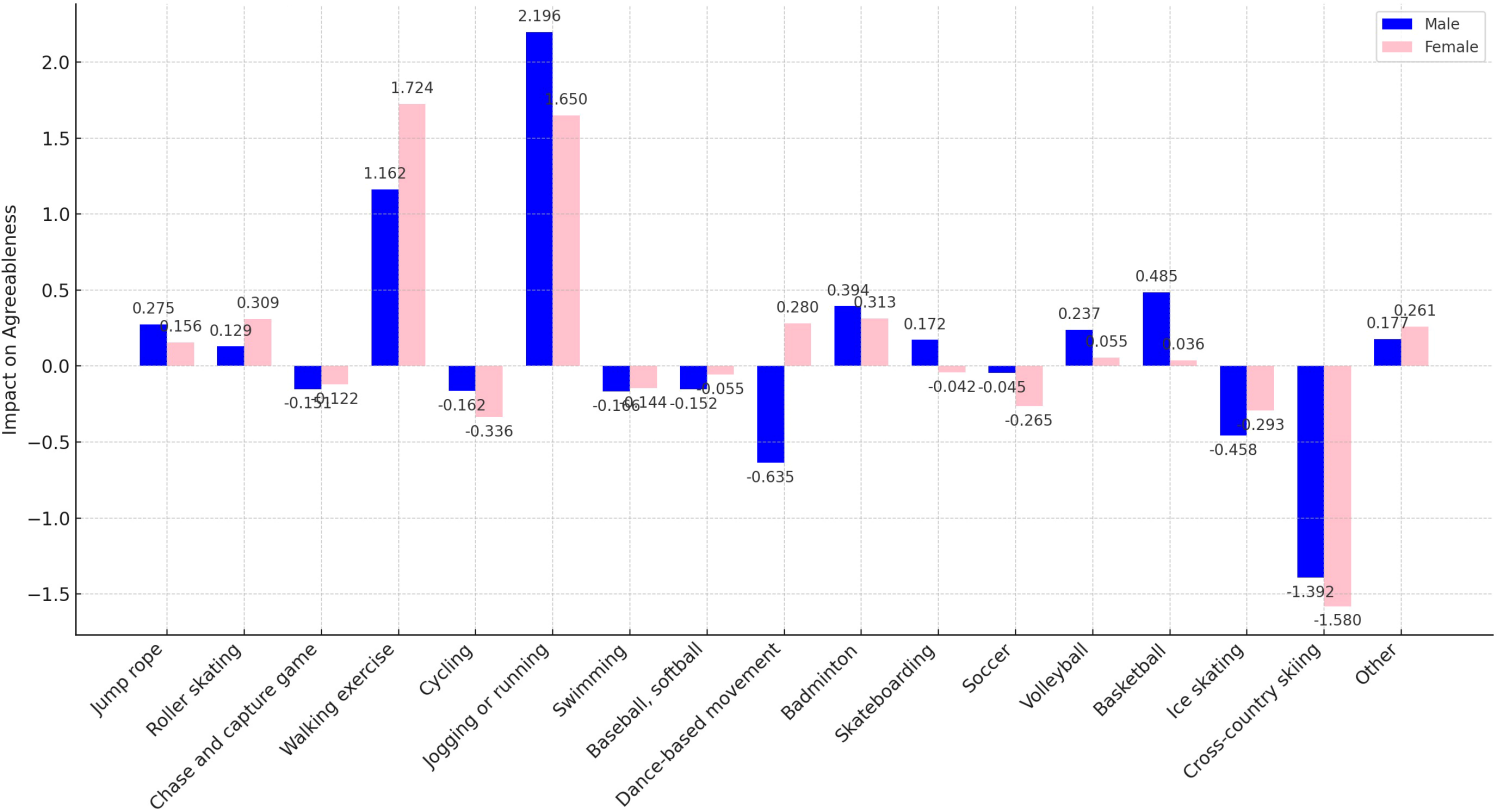
Impact of participation in different sports on agreeableness by gender.

**Figure 11:**
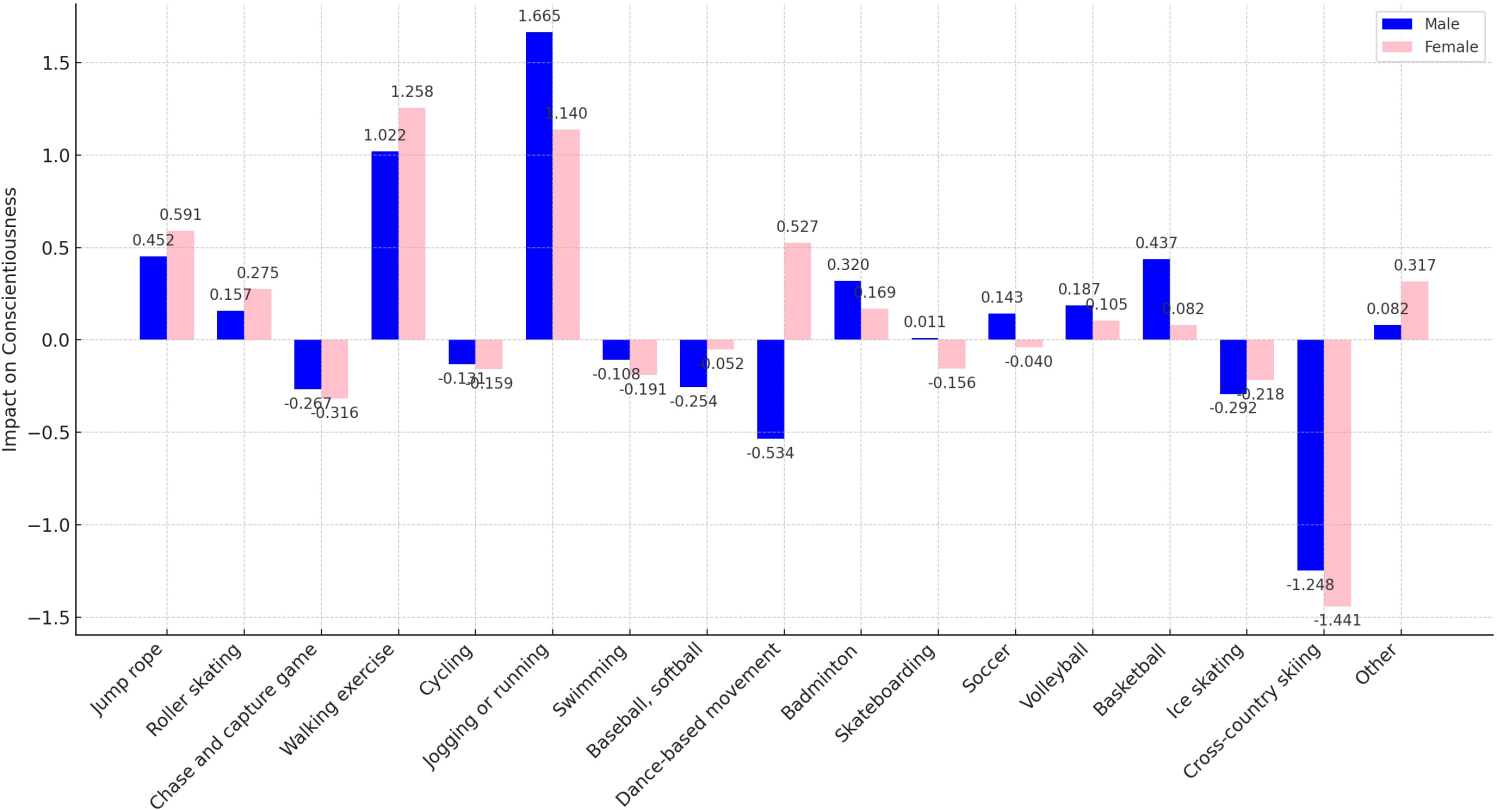
Impact of participation in different sports on conscientiousness by gender.

## 4 Discussion

### 4.1 Outstanding performance in walking exercises and jogging or running

Significant variations exist in how different sports affect personality traits, with walking and jogging or running notably effective in fostering such traits. Specifically, jogging or running greatly improves extraversion, agreeableness, and conscientiousness. In contrast, walking is particularly effective at mitigating neuroticism and boosting openness. These outcomes can be interpreted through several psychological lenses. Firstly, these activities enhance self-efficacy as participants can concretely measure their progress, boosting confidence in their capabilities and thereby their self-efficacy (Vassell, 2023). Increased self-efficacy encourages greater social interaction, which enhances extraversion (Esfandagheh et al., 2012). Secondly, these sports involve setting precise, achievable goals (Consolo, 2007). Achieving these goals not only improves organizational and persistence skills, fostering conscientiousness (Weinberg and Butt, 2014), but also enhances self-esteem and positive self-perception, indirectly promoting extraversion and agreeableness (Cetinkalp, 2012; Extremera et al., 2016; Lochbaum et al., 2016). Additionally, the interaction between physical activity and personality traits suggests that certain traits might predispose individuals to prefer certain sports (Kuper et al., 2023), which in turn might amplify those traits. For instance, extroverts may gravitate toward group sports like jogging clubs, which can further augment their extraversion (Asquith et al., 2022). This dynamic illustrates the complex interplay between personality and physical activity, highlighting how specific physical activity can differentially impact personality development.

From a physiological standpoint, the distinct impacts of walking exercises and jogging or running can be attributed to several factors: Firstly, endorphins and emotional regulation. These aerobic activities are known to enhance the release of endorphins (Kpame and Richard, 2020), neurotransmitters linked to improved mood and increased pleasure, which can significantly alleviate pain and enhance emotional well-being (Niikura et al., 2008; Jensen et al., 2017; Pilozzi et al., 2020). This boost in endorphins can lead to reduced stress and anxiety, thereby fostering positive personality development (Khoury and Nagy, 2023; Ye and Liu, 2023). Secondly, brain structure and function alterations. Regular aerobic exercise is beneficial for the growth of brain areas crucial for emotional regulation and cognitive function, notably the hippocampus and prefrontal cortex (Manning and Steffens, 2018). These adaptations can stabilize emotions and decrease neurotic tendencies. Thirdly, improved circulation and oxygenation. Activities like walking and jogging enhance cardiopulmonary efficiency, which boosts cerebral blood flow and nutrient delivery. This, in turn, supports neuronal health, crucial for enhancing traits such as openness, associated with creativity and novel experiences (Leasure and West, 2016). Fourthly, sleep quality enhancement. Consistent physical activity, such as walking and jogging, has been shown to improve sleep quality (Xu et al., 2023; Kline, 2014), a vital component for maintaining emotional balance and cognitive clarity, which indirectly supports the development of positive personality traits like conscientiousness and lowers neuroticism (Bender and Lambing, 2024; Semplonius and Willoughby, 2018). Fifthly, stress management. These exercises reduce stress reactions, lower cortisol levels, and bolster stress resilience (Ilmi et al., 2022; Ibrahim et al., 2020), contributing to increased conscientiousness and agreeableness and decreased neuroticism (Kaiseler et al., 2012; Mccrae, 1990).

From an evolutionary psychology perspective, walking and running were essential for survival in early human societies (Rolian et al., 2009; Nenko et al., 2018). Walking served not only as the primary method for locating food, water, and shelter but also played a crucial role in social interactions and the exploration of new territories. Running, particularly endurance running, was critical during hunting and when evading predators. Engaging in these activities today may trigger deep-seated psychological and physiological mechanisms that were developed to navigate these ancient challenges, contributing positively to the development of personality traits. This suggests that by participating in walking and running, modern individuals may tap into these evolutionary adaptations, which can enhance traits such as resilience, social connectivity, and exploratory behavior, ultimately supporting the positive evolution of personal characteristics.

### 4.2 Gender differences in the effects of sports participation on personality development

Our analysis reveals distinct gender-based preferences in sports that are optimal for the development of certain personality traits: men benefit more from jogging or running, particularly for enhancing neuroticism, agreeableness, and conscientiousness, while women gain more from walking exercises. In contrast, there are no gender differences in the optimal sports for boosting extraversion and openness, with both jogging or running, and walking exercises serving as effective activities. This pattern aligns with gender role theory, which posits that societal expectations around emotional expression and behaviors differ for men and women, influencing their personality development (Diekman and Schmader, 2024). Sports, as a form of behavior, are influenced by these gender roles. Typically, men are encouraged to participate in intense sports, while women are steered toward gentler activities. Meeting these societal expectations then triggers feedback from society, fostering personality development (Gantz and Wenner, 1991). By integrating our findings with gender role theory, we offer a fresh perspective on how gender roles through sports participation shape individual personality traits. This not only reaffirms the relevance of gender role theory in contemporary society but also broadens its scope, particularly in explaining gender-specific impacts of sports on personality traits. Moreover, our study underscores the importance of considering gender roles in the choice of sports and their potential influence on personality development, providing a solid empirical foundation for further research into gender role theory.

Our findings can also be interpreted through the lens of evolutionary psychology. Historically, men and women performed distinct roles within early human societies. Men primarily engaged in hunting and group protection (Panter-Brick, 2002), activities that involve chasing and quick movements, hence making jogging or running a natural fit for enhancing traits that were beneficial in these contexts. This evolutionary backdrop explains why these activities are particularly suited to men today. Conversely, women in ancient times were more engaged in gathering (Panter-Brick, 2002), an activity that involves walking and careful selection, aligning closely with the nature of walking exercises. This historical alignment explains why walking is an optimal activity for women. Even though modern lifestyles differ vastly from those of our ancestors, the adaptive traits ingrained in our genetic makeup continue to influence our sports preferences and how these activities mold our personalities. The distinct sports preferences between genders and their differential impact on personality traits can be viewed as modern manifestations of these ancient adaptive behaviors. This perspective not only enriches our understanding of gender-specific sports preferences but also highlights how deep-rooted evolutionary patterns continue to shape behaviors in contemporary settings.

The observed results may also be associated with the differing emotional regulation strategies employed by men and women. Various studies indicate that the ways individuals cope with stress significantly influence their personality development (Shiner, 2009; Pérez-Chacón et al., 2023; Schlatter et al., 2022). Men, in particular, are more inclined to use high-intensity sports as a means to regulate their emotions (Piekarska and Martowska, 2020). This preference for vigorous physical activities as a coping mechanism is a significant factor contributing to the gender differences in selecting optimal sports activities. This insight not only highlights the role of sports in emotional management but also sheds light on how traditional gender roles may influence the selection of sports as a tool for psychological well-being.

### 4.3 Adverse effects of cross-country skiing on personality traits

The earlier empirical analysis revealed that cross-country skiing notably and negatively influences all personality traits. Moreover, among sports that detrimentally affect personality trait development, the negative impact coefficients associated with cross-country skiing are consistently the most pronounced. This pattern persists even when accounting for other control variables. Therefore, it suggests that cross-country skiing should be cautiously considered or possibly avoided for those seeking to enhance any aspect of their personality positively. This recommendation is based on its consistent association with adverse outcomes in personality development, making it less suitable for those aiming to foster positive psychological growth.

The relationship between cross-country skiing and personality traits can be understood through several lenses: First, Psychological Stress: Cross-country skiing depends heavily on variable environmental factors such as snow quality and weather conditions, which introduce significant risks. Participants’ awareness of these risks can lead to increased fear and anxiety (Raue et al., 2019), accumulating stress that adversely impacts long-term psychological health and negatively influences personality traits (Frenkel et al., 2019). Second, Skill Requirements: Cross-country skiing demands extensive learning and high skill levels, involving challenges like endurance and technical prowess. The intense challenges and potential frustrations encountered can negatively affect personality traits by undermining individuals’ confidence and satisfaction (Taylor et al., 2022). Third, Social Interaction: The sport often lacks social interaction, focusing instead on solitary engagements with nature. This isolation can restrict social skill development, inhibiting growth in traits like agreeableness (Leep Hunderfund et al., 2022). Fourth, Physical and Emotional Exhaustion: The physical demands of cross-country skiing require significant endurance and strength, leading to exhaustion. This continuous strain can heighten susceptibility to mood disorders such as anxiety and depression, fostering a more neurotic disposition (Broddadóttir et al., 2021; Sosnowska et al., 2019). Fifth, Cultural Factors: While the sport can confer community recognition and status (Ballman et al., 1981), primarily participating to fulfill external expectations rather than for personal fulfillment can increase stress and negatively impact personality development (Weinstein and Ryan, 2011). This multifaceted perspective highlights how cross-country skiing’s demanding nature can shape personality traits, often posing challenges that may hinder positive development.

## 5 Practical value

By thoroughly examining the role of gender differences in the relationship between sports participation and personality development, we can gain a deeper understanding of the broad impact of sports on individual growth, thereby supporting efforts to create a healthier and more balanced society.

At the policy level, our findings can provide valuable insights for policymakers in the fields of education and public health. By elucidating the relationships between gender, exercise frequency, sports disciplines, and personality traits, this research can assist policymakers in formulating more inclusive and targeted sports promotion strategies. For instance, educational authorities might tailor physical education curricula and exercise schedules to address the distinct developmental needs of male and female students, thereby enhancing their personality development.

On a practical level, our results offer important guidance for sports coaches, psychological counselors, and sports enthusiasts. Recognizing how gender, exercise frequency, and participation in different sports disciplines influence personality development can help practitioners design more personalized training and development plans that account for gender-specific factors. This approach could more effectively foster the physical and mental well-being, as well as the positive personality development, of both athletes and the general population. Furthermore, this research provides fresh avenues for interdisciplinary studies in the fields of sports science and psychology, encouraging collaborative exploration of these topics.

## 6 Limitations and future research directions

### 6.1 Limitations

This study’s sample, consisting of middle and high school students from 17 cities within Shandong Province, while sizable, is regionally confined, potentially limiting the findings’ applicability and generalizability. Cultural and economic differences across regions, along with varying levels of engagement in physical activities, could differentially impact adolescent personality traits.

In terms of analytical methods, this study employs the seemingly unrelated regression model. Although this model accounts for the correlations between dependent variables and improves model efficiency, it is still based on the assumption that all observations are independent of each other. This assumption may not hold, especially when the sample exhibits clustering based on unobserved characteristics. Additionally, the data used in this study are cross-sectional, making it impossible to establish causal relationships between variables, which limits our understanding of how participation in sports influences personality traits over time. Finally, due to limitations of the database itself, some confounding variables that might affect both sports participation and personality traits could not be controlled for in the analysis, which limits the certainty of the study’s findings.

### 6.2 Future research directions

Building on the insights from this study, future research can take several paths to deepen our understanding of how sports influence adolescent personality development and explore ways to harness sports for positive personality growth.

Firstly, although we found that walking and jogging/running were prominent in promoting the development of personality traits, the psychological mechanisms behind this effect were not analyzed. We hope that future research will focus on empirical analysis of these psychological mechanisms to better understand the complex relationship between physical activity and personality traits. Secondly, more granular studies on different sports types are needed. While this research has considered various sports, detailed analyses on how specific sports and their intensity, social aspects, and skill demands influence personality traits are still required. Such studies can help identify which sports are most beneficial for certain personality traits. Lastly, further investigation into gender differences is essential. Our findings highlight gender as a key factor affecting how sports influence personality traits. Future studies should explore the underlying mechanisms of these gender differences more comprehensively, incorporating physiological, psychological, and sociocultural dimensions. Research could include examining how different genders experience sports psychologically and how these experiences shape personality development. These avenues promise to enrich our understanding of sports as a developmental tool and tailor approaches that leverage physical activity to foster desirable personality traits in youths.

## Data Availability

The raw data used in this study, as well as the Stata code for processing the data, are available upon request from the corresponding author. Access to raw data may be subject to restrictions based on e.g., ethical considerations, confidentiality agreements.
